# Causes of death in patients with locally advanced head and neck cancer treated with radiotherapy and systemic therapy

**DOI:** 10.1186/s12885-019-6427-1

**Published:** 2019-12-21

**Authors:** I. Zapata, M. Alvarez, R. Hidalgo, B. Pajares, M. J. Garcia-Anaya, M. D. Toledo, J. M. Trigo, Y. Lupiañez-Perez, J. A. Medina, J. Jaime Gomez-Millan

**Affiliations:** 1 Radiation Oncology Department, Hospital Universitario Virgen de la Victoria, Campus Teatinos s/n., 29010 Malaga, Spain; 2 Laboratorio de biología molecular del cancer, centro de investigaciones medico-sanitarias (CIMES), Universidad de Málaga, Malaga, Spain; 3 Instituto de investigación biomédica de Malaga, Campus Teatinos s/n., 29010 Malaga, Spain; 4 Pathology Department, Hospital Universitario Virgen de la Victoria, Campus Teatinos s/n., 29010 Malaga, Spain; 5 Servicio central de Informatica, Universidad de Malaga, Malaga, Spain; 6 Medical Oncology Department, Hospital Universitario Virgen de la Victoria, Campus Teatinos s/n, 29010 Malaga, Spain

**Keywords:** Competitive mortality, Non-tumoral death, Head and neck cancer

## Abstract

**Background:**

To investigate the incidence of non-cancer mortalities and prognostic factors associated with competitive causes of death in a homogeneous cohort of patients with locally advanced head and neck cancer treated with radiotherapy and systemic treatment.

**Methods:**

This study included 284 patients with locally advanced head and neck cancer treated with radiotherapy and systemic treatment between 2005 and 2017. The cumulative incidence of death associated with tumour, second tumours, treatment, side effects and comorbidity was calculated. A Fine and Gray regression model was used to investigate factors associated with cancer and competitive mortality.

**Results:**

The cumulative incidence of tumoral death at 5 and 10 years were 35 and 47% respectively, whereas the cumulative incidence of competitive mortality were 10 and 12% respectively. In the multivariate analysis, age and comorbidity were independent factors for non-cancer mortality. Patients with a high risk of non-cancer mortality presented a cumulative incidence of 17.3% at 5 years and 18.4% at 10 years.

**Conclusions:**

This study demonstrated a high incidence of competing mortality in older patients with comorbidities. Non-cancer deaths should be considered when selecting patients for combination therapies and in the study design ofclinical trials.

## Background

Patients with locally advanced head and neck cancer (LAHNC) treated with combinations of radiotherapy (RT) and chemotherapy (CT) may experience higher incidence of competitive (non-tumoral) mortality compared with the healthy population [1], and a decrease in the therapeutic ratio of treatment.

The major causes of competitive mortality in LAHNC are comorbidities, toxicities associated with treatment regimens, and second primary tumours. Survivors of LAHNC present an increased incidence of comorbidities associated with alcohol and tobacco consumption, and maybe predisposed to an increased risk of a non-tumoral cause of death [2]. Different studies have shown that comorbidity is an independent prognostic factor for survival in head and neck cancer [1, 3–6]. Moreover, aggressive treatment with RT and CT has been associated with a significant number of acute and chronic complications, which are potentially life-threatening, causes the therapeutic ratio to decrease and limit the potential benefit of the treatment [7, 8]. Finally, field cancerization resulting from exposure to tobacco and alcohol predisposes the development of second primary tumours [9], which are a major cause of mortality in LAHNC survivors [10].

In recent years, intensity modulated radiotherapy (IMRT) has been shown to decrease acute and chronic toxicity in head and neck cancer patients compared with 3D conformal radiotherapy (3D-RT) or 2D radiotherapy (2D-RT). Randomised studies have not shown an increase in survival in these patients [11], however, some recent studies point to a potential increase in survival among patients treated with IMRT, which might be attributed to a lower toxicity [12].

Different authors have investigated several patient prognostic factors to identify predictors of competitive mortality in head and neck cancer [1, 13–15]. However, most of these studies investigated factors related to the patient, with only one study including factors related to treatment regimens and RT techniques [15]. In this study, Mulcahy et al. included patients with larynx cancer, treated with total laryngectomy in a 42% of cases (230 patients), and without systemic treatment in 83% (191 patients),. In the present manuscript we aim to investigate the incidence of non-cancer mortalities and prognostic factors associated with competitive causes of death, not only including factors related to the patient but also to the treatment, such as treatment technique, therapeutic regimen used and associated side effects, in a homogeneous cohort of patients with locally advanced head and neck cancer who received RT and systemic treatment.

## Methods

### Patients and treatments

A total number of 284 patients treated under different successive institutional protocols from January 2005 to April 2017 were included in the analysis. The selection criteria were a diagnosis of squamous carcinoma of the oral cavity, oropharynx, hypopharynx and larynx, American Joint Committee on Cancer stage III-IV [16]. Comorbidity was evaluated with the cancer-specific comorbidity index for head and neck cancer (CCI-HN) published by Boje et al. [6]. The vast majority of the patients (96%) were current smokers.

All patients were treated with a curative intention, receiving combinations of radiotherapy and chemotherapy or cetuximab. Radiotherapy was given with different dose-fractions, such as daily fractions of 1.8 Gy with an extra second fraction to the boost volume during the last 13 fractions, with a total dose of 69.9 Gy (concomitant boost) [17], daily fractions of 2 Gy with a total dose of 70 Gy (conventional) [18], and daily fractions of 2.17 Gy, with a total dose of 65.10 Gy to the high dose planning target volume (PTV), and fractions of 1.8 Gy with a total dose of 54 Gy to the low dose PTV (simultaneous boost). Planning was performed with 3D-RT in the concomitant boost and conventional group, and IMRT in the simultaneous boost group.

Different systemic treatments were combined with RT. Concomitant chemoradiotherapy (CRT) consisted of CDDP with doses of 100 mg/m2 every 3 weeks combined with RT (conventional or simultaneous boost), or CDDP with doses of 40 mg/m2 every week in patients treated with concomitant boost. The combination of RT and cetuximab (RTCTX) consisted in the infusion of cetuximab with an initial loading dose of 400 mg/m2, and weekly doses of 200 mg/m2 combined with RT.

Induction chemotherapy (ICT) consisted of CDDP with doses of 100 mg/m2 day 1 and 5-Fu 1 g/m2 in continuous infusion, days 1–5 of every cycle, every 21 days for a total of 3 cycles (PF), or CDDP to doses of 75 mg/m2 day 1, 5-Fu 750 mg/m2in continuous infusion days 1–5, and docetaxel 75 mg/m2 day 1, 1 cycle every 21 days for a total of 3 cycles (DPF). After induction chemotherapy, RT was planned with 3D-RT (PF) or IMRT (DPF), combined with cetuximab, with an initial dose of 250 mg/m2, and weekly doses of 200 mg/m2, or CDDP with doses of 100 mg/m2 every 3 weeks.

Competitive mortality was defined as the time to death for any cause in patients with a controlled tumour. Acute and chronic toxicity were evaluated with the common terminology criteria for adverse events (CTCAE, v4.0) [19] and the RTOG chronic morbidity score [20] respectively. To consider the toxicity of larynx and mucositis in combination, an item called laryngo-pharynx toxicity, which considered larynx or pharynx toxicity, was created.

Causes of death were considered as follows: death associated with acute toxicity was considered as death during the treatment or in the first 90 days after the end of the treatment, without tumoral progression. Death associated to chronic toxicity was considered as death 90 days or more after the end of the treatment, without tumoral progression. Death associated to comorbidity was defined as a non-treatment related, intercurrent death in patients without active disease. Death related to secondary tumour was considered a tumoral death.

### Follow up

Patients were evaluated 3 months after the completion of treatment with a physical examination, and computed tomography or MRI of the head and neck region, and chest X- Ray. Response was evaluated following RECIST 1.1 criteria (Response Evaluation Criteria in Solid Tumours) [21]. Thereafter, patients were evaluated every 3 months in the first and second year, every 6 months during third, fourth and fifth year, and annually thereafter. A routine computed tomography was obtained in every evaluation during the first, second and third year, and yearly thereafter. Other tests such as magnetic resonance imaging, endoscopy and biopsies, were selectively used based on the clinical situation. All recurrences were biopsy proven. Follow-up continued until April 2017.

### Statistical analysis

Causes of death were analysed with a competitive risk analysis. We defined a death as associated with comorbidity when the patient died without evidence of a tumour, due to a non-tumoral disease. Competing risk was defined as the different events that either hinder the observation of the event of interest or modify the chances that this event will occur [22]. The method to analyse competing risk is based on cumulative incidence function, which is estimated both for the event of interest and for all competing events, and their estimates depend on each other. The cumulative incidence (CIR) from tumoral and competitive causes was calculated using non-parametric cumulative incidence functions. The comparison of cumulative incidence between groups was performed using the Gray test [23]. For the multivariate analysis of cumulative incidence, a semi-parametric model for subdistribution hazards was performed as proposed by Fine and Gray [24]. Covariates with *p* < 0.1 in the univariate analysis were included in the multivariate model. A *p*-value of < 0.05 was considered statistically significant.

From the multivariant competitive risk regression model we obtained a risk score for each patient for tumoral and non-tumoral death [25]. The mean score was calculated and used as a cut-off point, and patients were divided into low or high risk depending on the final score obtained. Statistical analysis was performed with IBM SPSS 24.0 software [26], and the CIR functions of competitive death, considered as competing risk, with R software [27].

## Results

### Patient characteristics and treatment

Overall patient characteristics are summarised in Table 1. Most of the patients were men, with a median age of 60 years, stage IV, and had larynx tumours. The majority of patients presented an ECOG of 0–1 (ECOG scale of performance status), with only 5% presenting an ECOG 2. The predominant tumour location was the larynx, with 47% of the patients presenting this tumour site. The most frequent comorbidity was chronic pulmonary disease, that was present in 20% of patients (Table 2).

**Table 1:** Patient and treatment characteristics

	Number of patients (*n* = 284)	Percentage of patients (%)
Age		
Median (range)	60 (34–84 a)	
Sexo		
Male	256	89%
Female	28	11%
ECOG		
0	164	58%
1	104	37%
2	16	5%
Cormorbidities (CCI-HN)		
0 = No comorbidity	142	50%
1 = Mild	90	32%
2 = Moderate	45	16%
≥ 3 = Severe	7	6%
Localization		
Oral cavity	30	11%
Oropharynx	82	29%
Larynx	134	47%
Hypopharynx	38	13%
Stage		
III	103	36%
IVA	144	51%
IVB	37	13.0%
T Stage		
T1	13	5%
T2	44	15%
T3	142	50%
T4	84	30%
N Stage		
N0	96	34%
N1	52	18%
N2	113	40%
N3	23	8%

**Table 2:** Incidence and distribution of comorbidity

Comorbidity	Patients	Percentage of total patients (%)
Congestive heart failure	17	6%
Cerebral vascular disease	21	7%
Gastric ulcer	22	7%
Diabetes	38	16%
Liver disease	51	18%
Chronic pulmonary disease	58	20%

Regarding the treatment characteristics (Table 3), most patients were treated with CRT (56%), and the most frequent treatment planning technique was 3D-RT (74%). Patients were treated with RT and different regimens of systemic treatment. A total of 160 patients (56%) were treated with CRT, 73 patients (26%) with RTCTX, and 51 patients with ICT (18%). Of the total of patients treated with ICT, 31 patients (52.5%) were treated with ICT followed by RTCTX, and 28 patients (47.5%) were treated with ICT followed by CRT.

**Table 3:** Variables related to treatment

	Patients (n = 284)	Percentage (%)
Technique		
3D-RT	211	74%
IMRT	73	26%
Fractionation		
Conventional	143	50%
Concomitant boost	74	26%
Simultaneous boost	67	24%
Treatment regimen		
CRT	160	56%
RTCTX	73	26%
ICT	51	18%

*CRT* Chemoradiotherapy, *RTCTX* Radioterapy/cetuximab, *ICT* Induction chemotherapy, *IMRT* Intensity modulated radiotherapy, *3D-RT* Three dimensional radiotherapy

With respect to acute toxicity, a total of 47 and 35.4% of patients presented acute > G2 symptomatic mucositis and larynx toxicity respectively, and 83% of the patients showed acute G > 2 laryngo-pharynx toxicity. On the other hand, 27% presented chronic symptomatic mucositis, 6% of the patients suffered from chronic laryngeal toxicity, and 9% suffered from chronic G > 2 pharyngo-laryngeal toxicity (Table 4). The acute mucositis associated with the different fractionation was observed as follows: In patients treated with conventional fractionation presented G0–2 mucositis in 64% (92 patients), and 36% (51 patients) presented ≥ G3 mucositis. Patients treated with concomitant boost presented G0–2 mucositis in 42% (31 patients), and 58% (43 patients) presented ≥ G3 mucositis. Finally, patients treated with simultaneous boost presented G0–2 mucositis in 40% (27 patients), and 60% (40 patients) presented ≥ G3 mucositis.

**Table 4:** Variables related to treatment toxicity

	Patients (*n* = 284)	Percentage (%)
Mucositis		
G0	27	9.5%
G1	37	13%
G2	86	30%
G3	127	45%
G4	9	3.5%
Acute pharynx-larynx toxicity		
G0	11	4%
G1	14	5%
G2	23	8%
G3	196	69%
G4	43	15%
Chronic pharynx-larynx toxicity		
G0	91	32%
G1	98	34.5%
G2	70	25%
G3	21	7.4%
G4	4	1.5%
Anemia		
G0–1	252	89%
G2–3	32	11%
Neutropenia		
G0–1	230	81%
G2–3	43	15%
G4–5	11	4%

### Outcomes

#### General outcomes

**Fig. 1:**
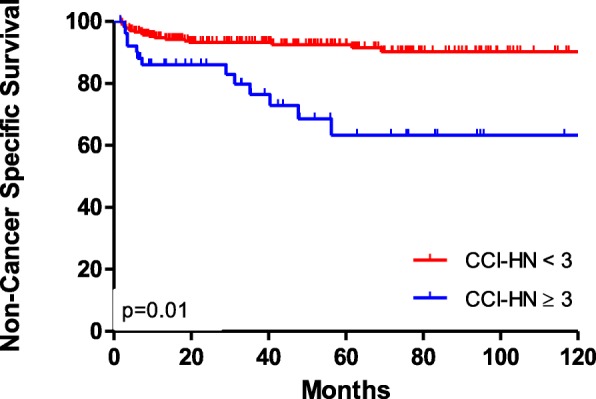
Non-cancer specific survival (Kaplan Meier) of patients with low and moderate-high comorbidity index

With a median follow up of 74 months, 5 and 10-year overall survival (OS) and progression-free survival (PFS) were 49 and 31%, and 35.5 and 22% respectively. Cancer specific survival (CSS) and Non-cancer specific survival (NCSS) were 62 and 49% at 5 and 10 years, and 89 and 86% respectively. Moreover, patients with a low comorbidity index presented a 5 year NCSS of 91% compared with 65% in patients with a moderate and high comorbidity index (*p* = 0.01) (Fig. 1). A significant association was shown between age and ECOG, with 56.6% of patients over 70 years presenting ECOG ≥1 compared with 40% in patients with less than 70 years (*p* = 0.04). Moreover, patients with less than 70 years were treated more frequently with CRT (72%) compared with older patients (30.5%) (*p* = 0.01). Furthermore, patients with ECOG 0 were more frequently treated with CRT (72.5%) than patients with ECOG ≥1 (56%) (*P* = 0.01). Finally, patients with a lower comorbidity score (CCI-HN 0–1) were more frequently treated with CRT (86%) compared with those who had higher comorbidity (CCI-HN 2–6) (74%) (*p* = 0.01).

#### Causes of death

During the study follow-up period, 153 patients died. Of those, 105 (69%) died as a consequence of the tumour, 15 (10%) as a consequence of a secondary tumour, and the deaths of 32 (21%) patients were related to non-tumoral competitive causes of death. Of these non-tumoral deaths, 14 patients (9% of the total) died as a consequence of acute toxicity, 4 patients (3%) died of chronic toxicity, 14 patients (10%) died of comorbidity, and 1 patient died from an unknown cause. The cause of death of 38 patients was associated with a second tumour. The most frequent tumours were LAHNC in 12 patients (30%), lung cancer in 10 patients (25%), digestive tumours in 7 patients (17%), urologic tumours in 7 patients (18%), skin cancer in 2 patients (4.5%) and hematological tumours in 2 patients (4.5%).

**Fig. 2:**
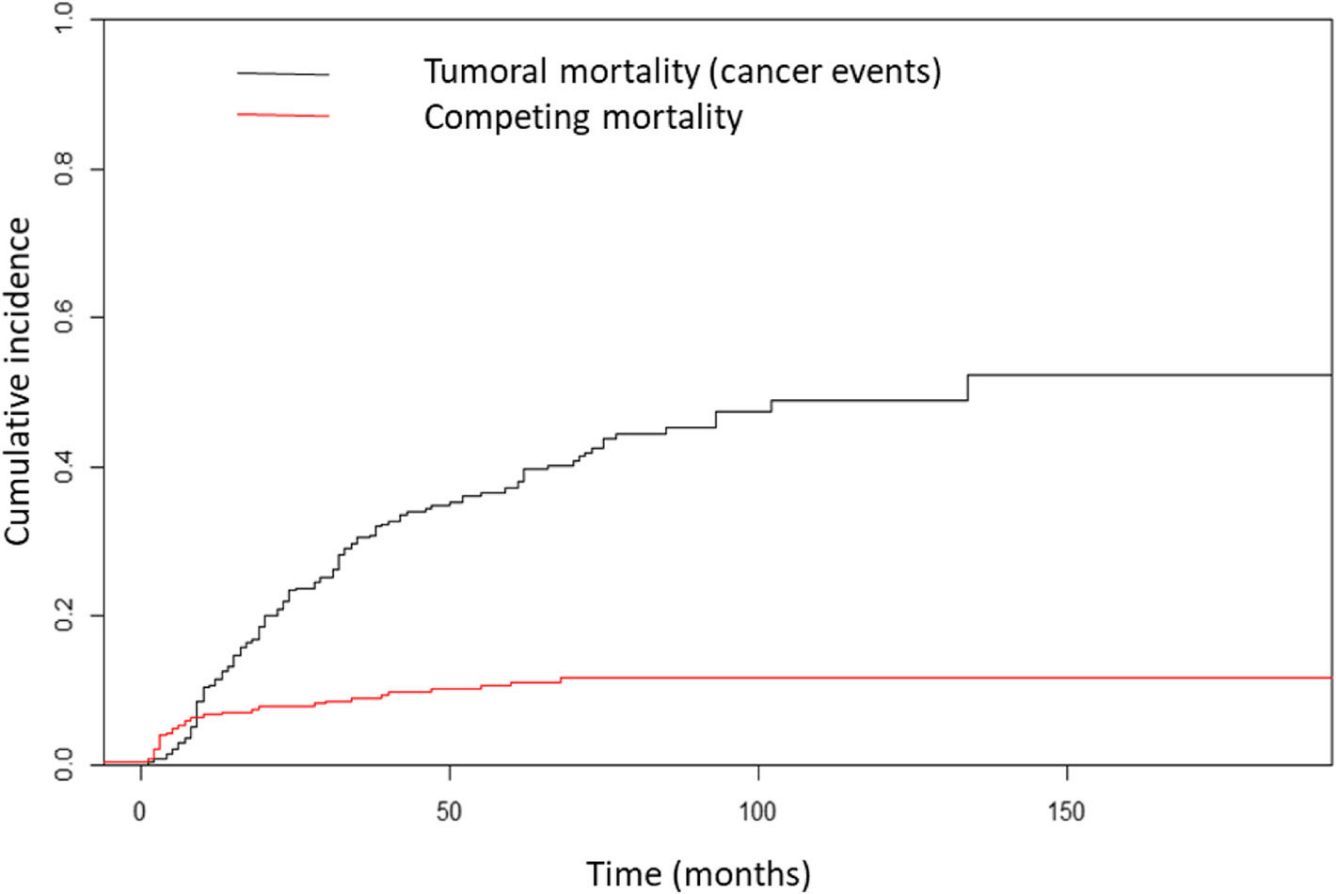
Cumulative incidence of cancer death and competitive causes of death of tumoral and non-tumoral causes

With a median follow up of 74 months, the CIR of tumoral death at 5 and 10 years was 35% (CI 95% 29–40) and 47% (CI 95% 40–54), respectively. On the other hand, the CIR of competitive mortality was 10% (CI 95% 7–14) and 12% (CI 95% 8–16) respectively (Fig. 2). Of the total of competitive deaths, the 5-year CIR of death associated with comorbidity, acute and chronic toxicity was 4.1, 4.6 and 1.3% respectively.

#### Factors associated with competitive and tumoral mortality

**Fig. 3:**
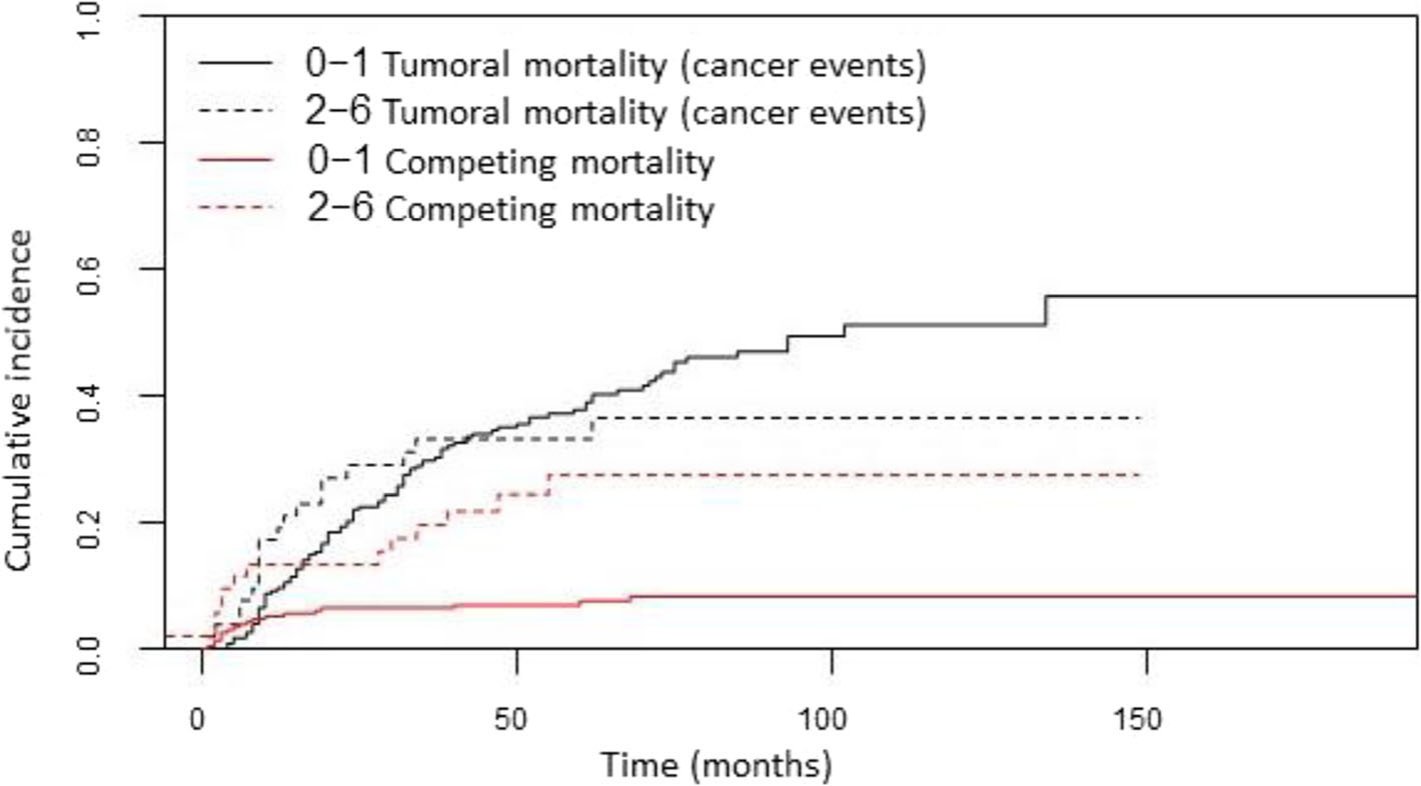
Cumulative incidence of cancer death and competitive causes of death in patient with low or absence (0–1) of comorbidity and moderate to high (2–6) comorbidity

Regarding the causes of competitive mortality, the univariate analysis showed that comorbidity presented significant differences in the CIR of death**.** Patients with low comorbidity presented a CIR of non-tumoral death of 7 and 8.3% at 5 and 10 years respectively, compared with a CIR of 24.6 and 27.6% in cases with moderate-high comorbidity (*p* = 0.01) (Fig. 3). Moreover, patients with low acute mucosal toxicity showed a CIR of non-tumoral death of 7.4 and 8% at 5 and 10 years, compared with 13 and 16% for cases with high toxicity (*p* = 0.07) (Table 5). After adjustment using the multivariate model, age as a continuous variable (HR 1.03; CI 95% 1–1.08; p 0.06), and comorbidity (HR 3.42; CI 1.46–7.97; *p* = 0.01) were significantly associated with competitive mortality (Table 6).

**Table 5:** Five year cumulative incidence of cancer and competitive mortality

Covariate	Cancer events			Competitive mortality		
	Cumulative Incidence	95% CI	p	Cumulative Incidence	95% CI	p
Sex						
Male	40.4	34.0–46.7	0.3	11.0	7.7–16.0	0.48
Female	33.3	13.6–54.6		14.0	4.3–29.2	
Comorbidity						
0–1	35.50	29.2–41.8	0.29	7.0	4.6–11.7	0.01
2–6	33.30	20.7–46.4		24.6	17.9–46.2	
ECOG						
0	30.20	23.2–37.4	0.01	9.30	5.5–14.6	0.35
1–2	42.30	33.0–51.3		11.0	6.2–17.5	
Stage						
III	24.4	16.6–33.1	0.01	11.7	6.4–18.9	0.21
IV	41.8	34.1–49.1		9.0	5.4–13.9	
Location						
Oral Cavity	41.0	17.8–63.5	0.35	10.0	2.5–23.9	0.99
Oropharynx	39.0	27.8–49.8		13.0	6.6–21.9	
Larynx	37.0	28.2–45.5		11.0	6.3–17.4	
Hypopharynx	52.0	32.6–68.6		10.0	3.2–22.2	
Treatment						
ICT	52.00	36.3–65.6	0.01	3.92	0.7–12.0	0.63
CRT	27.10	20.3–34.3		10.1	6.6–16.6	
RTCTX	41.60	30.0–52.7		12.4	6.0–21.1	
RT Technique						
3DRT	40.0	33.0–46.4	0.8	12.5	8.4–17.4	0.4
IMRT	58.0	17.9–84.0		8.0	2.7–17.8	
RT Fractionation						
Conventional	37.0	29.1–45.4	0.63	12.0	7.2–17.8	0.66
Others	43.0	33.7–52.5		12.0	6.5–18.6	
Acute PL toxicity						
0–1	28.1	15.9–41.6	0.5	9.0	2.7–19.3	0.34
≥ 2	36.6	30.2–42.8		10.3	6.8–14.7	
Acute mucosa toxicity						
0–2	36.4	28.6–44.2	0.66	7.4	3.9–12.5	0.07
3–5	34.0	25.8–42.3		13.0	7.9–19.5	
Chronic PL toxicity						
0–1	35.8	32.6–47.5	0.93	11.3	8.3–18.2	0.47
≥ 2	34.1	27.8–49.5		8.0	3.5–15.3	

*CI* confidence interval, *DFS* disease-free survival, *ECOG* Eastern Cooperative Oncology Group, *HR* Hazard ratio, *OS* Overall Survival, *PL* Pharyngolarynx

**Table 6:** Multivariate analysis of the cumulative incidence of cancer events and competing mortality

	Cancer events			Competing events		
Covariate	HR	95% CI	p-value	HR	95% CI	p-value
Age	0.99	0.97–1.01	0.6	1.03	0.1–1.08	0.06
Sex						
Male vs Female	0.59	0.28–1.22	0.15	1.74	0.55–5.47	0.34
Comorbidity						
0–1 vs 2–6	0.86	0.48–1.55	0.62	3.42	1.46–7.97	0.01
ECOG						
0 vs 1–2	1.50	1.01–2.21	0.04	0.94	0.42–2.1	0.88
Stage						
III vs IV	1.73	1.14–2.63	0.01	0.63	0.25–1.33	0.20
Technique						
3DRT vs IMRT	0.61	0.34–1.09	0.1	0.76	0.25–2.30	0.63
Treatment						
CRT, RTCTX,ICT	1.08	0.76–1.53	0.68	1.16	0.66–2.06	0.60
Acute Mucositis						
0–2 vs 3–5	0.80	0.51–123	0.3	1.63	0.72–3.68	0.24
Acute PL toxicity						
0–2 vs 3–5	1.33	0.79–2.23	0.29	1.24	0.44–3.48	0.69
Chronic PL toxicity						
0–2 vs 3–5	0.89	0.57–1.38	0.60	0.78	0.36–1.72	0.54

*CI* confidence interval, *ECOG* Eastern Cooperative Oncology Group, *HR* hazard ratio

On the other hand, in the univariate analysis, the factors associated with the CIR of tumoral death were ECOG, stage and treatment. Patients with an ECOG 0 presented a CIR at 5 and 10 years of 30.2and 43% compared with 42.3and 53% in ECOG 1–2(*p* = 0.01). Stage III patients presented a CIR of tumoral death at 5 and 10 years of 24.4 and 35.2% compared with 41.8 and 55.2% in stage IV respectively (*p* = 0.01). Patients treated with ICT presented a CIR of tumoral death at 5 and 10 years of 52 and 56.2%, CRT treated-patients presented a CIR of 27.1 and 39%, and RTCTX patients a CIR of 41.6 and 56% respectively(*p* = 0.01) (Table 5). After adjustment using a multivariate model, ECOG (HR 1.50; IC 1.01–2.21; *p* = 0.04) and stage (HR 1.73; 1.14–2.63; *p* = 0.001), were significantly associated with tumoral death (Table 6).

**Fig. 4:**
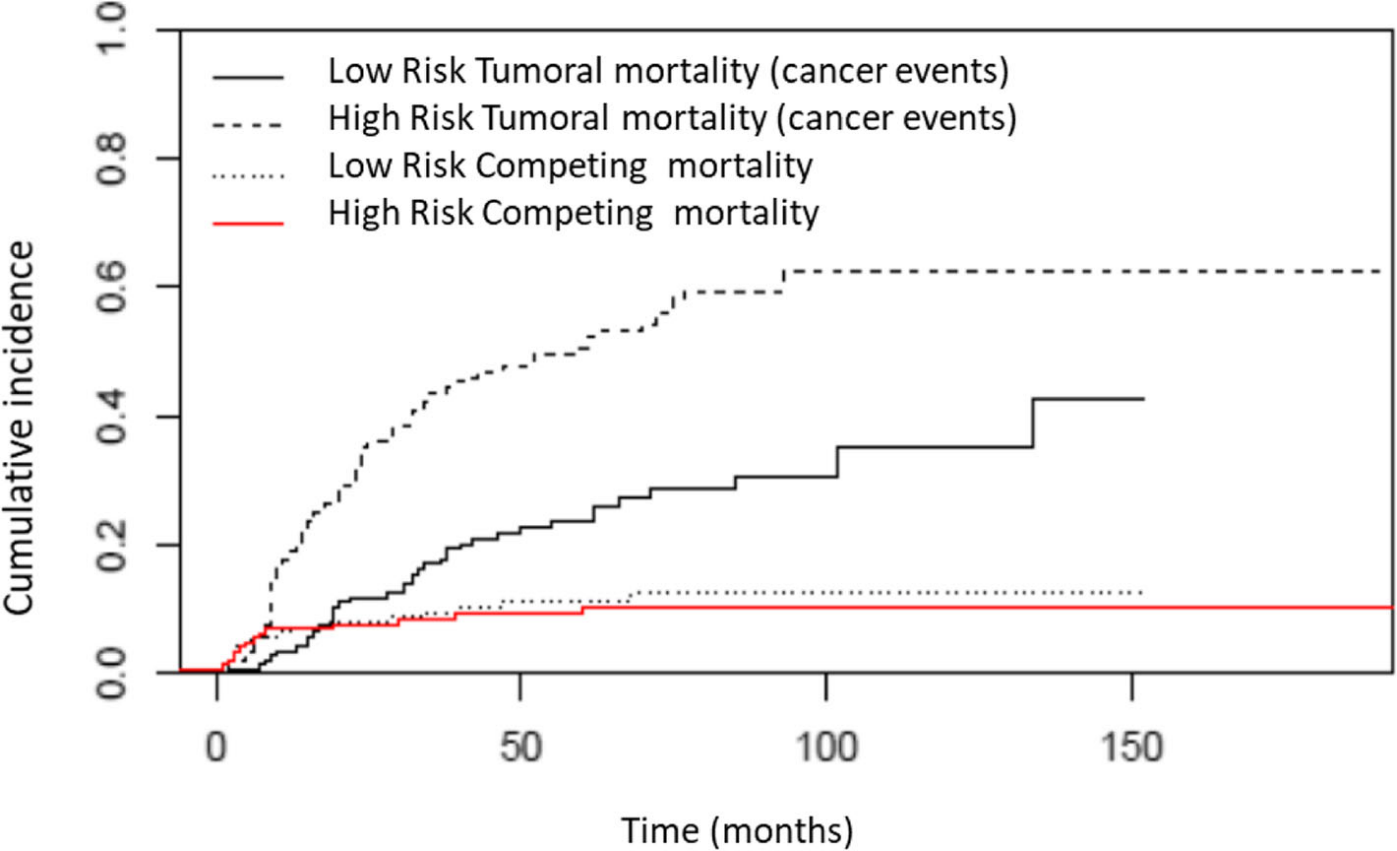
Cumulative incidence of cancer death in patients with high or low risk

**Fig. 5:**
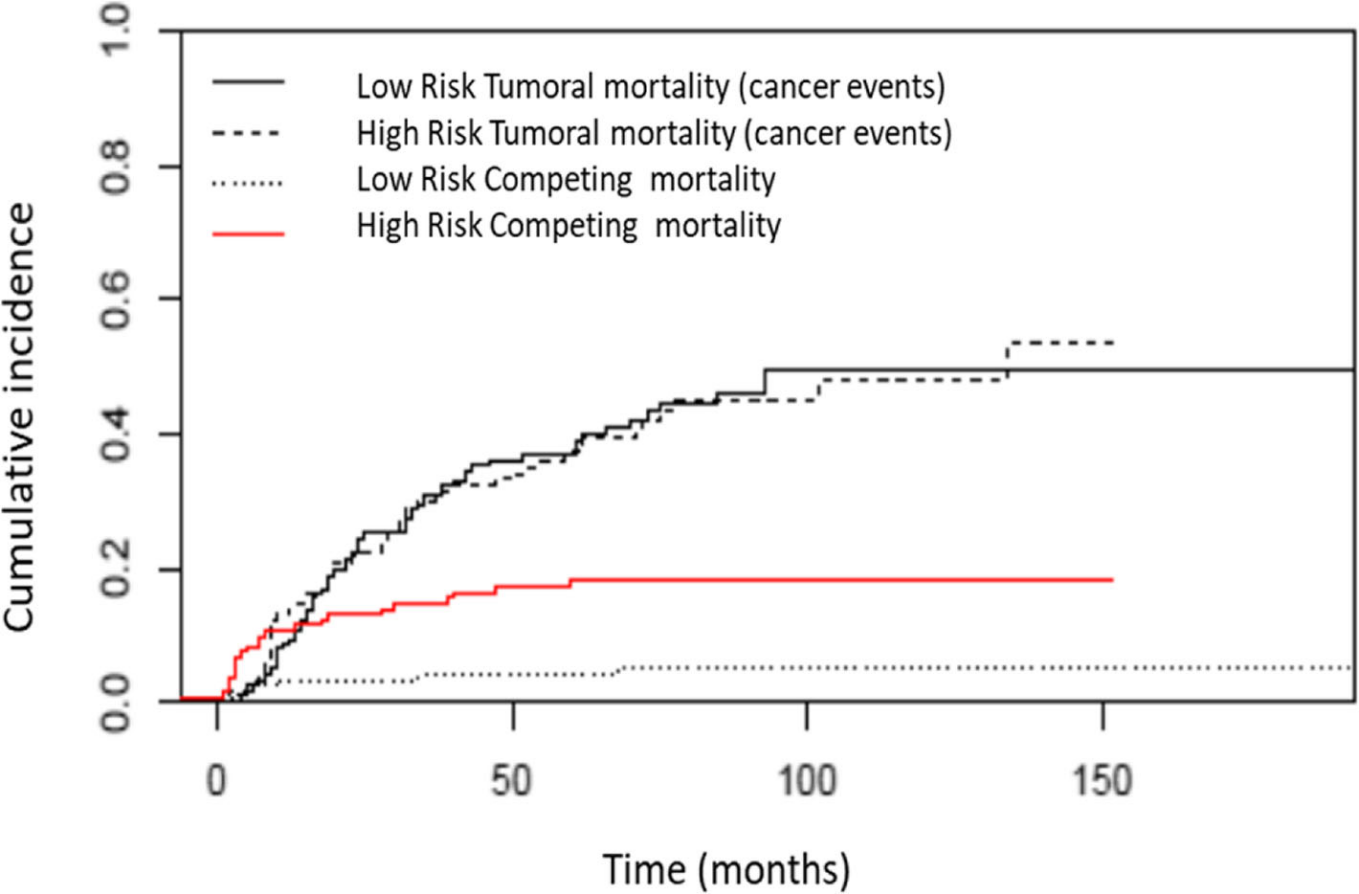
Cumulative incidence of competitive death in patients with high or low risk

Finally, patients were divided into high or low risk of competitive and tumoral mortality respectively. The CIR of low-risk patients presenting a tumoral death at 5 and 10 years was 22.7 and 30.5% respectively, whereas for high-risk patients it was 47.8 and 62.3% respectively (Fig. 4). On the other hand, the CIR for competitive mortality at 5 and 10 years was 4 and 5.1% for patients with low risk, and 17.3 and 18.4% respectively for high-risk patients (Fig. 5).

## Discussion

In this manuscript we have investigated the incidence of competitive mortality in a cohort of patients with LAHNC treated in our centre with different combinations of RT and CT or RTCTX. We found a 5-year incidence of non-tumoral death of 11.8%. These results are similar to those reported by Rose el al. in a study with more than 34,000 patients from the SEER database, which reported a CIR of 13%. However, other studies have reported a decreased rate of competitive and tumoral mortality, which presented an incidence of 8.9% and [28], and 6.5% [14] respectively. The lower incidence of deaths compared with our results might be explained by the different characteristics of the patients included in the studies, with a high number of resectable tumours treated with surgery and adjuvant RT or CRT [28], and of tumours in initial stages [17].

The association of age with causes of death has been thoroughly investigated in the literature [29]. Different studies have shown that patients older than 70 years do not benefit from combinations of RT and systemic therapy [29, 30]. Moreover, several studies have identified age as a significant risk factor for a non-competitive cause. Likewise, different authors have shown that age at 10 year intervals increased risk by 1.53 and 2.23 respectively [28, 31]. Furthermore, Takenaka et al. showed that an age of more than 75 years was associated with a higher incidence of a CIR of non-tumoral death (HR 2.59) [14]. We have shown that age is a significant prognostic factor for presenting a non-tumoral death, with a HR of 1.05 per year. As it has been consistently shown in the literature, age is a major prognostic factor that should be considered when adding a systemic agent is considered.

The impact of comorbidity on the survival of patients treated for LAHNC has been studied by different authors. In a recent study using a comorbidity index based on a modified Charlson index, Boje el al. showed in a multivariate model that patients with a high comorbidity index presented 1.9 more risk of dying for any cause [6]. These results have recently been confirmed by other authors, showing that patients with moderate-high comorbidity presented lower OS and cancer-specific survival [15]. Moreover, a recent publication [32] that prospectively analysed death associated to comorbidity in 190 patients treated with RT or CRT, reported a CIR of 4.4% at 5 years. Our results confirm these findings, showing that patients with moderate-severe comorbidity presented a risk of dying from a non-tumoral cause 3.4 times higher than patients with no or low comorbidity. These incidences are lower than those described by other authors, who found a cumulative incidence of 11% [13]. These differences might be explained by the different definitions of death associated with comorbidity. In our case, we considered death associated with comorbidity only in patients who had a complete response of the treated tumour.

We have shown that age and comorbidity are the two main predictive factors of non-tumoral death in LAHNC treated with RT and systemic therapy, with a CIR for high-risk patients of 17.3 and 18.4% at 5 and 10 years respectively. A recent paper confirms these findings, showing that age-adjusted CCI is a significant prognostic factor for non-tumoral death [15]. Moreover, these authors have shown that the risk of competitive mortality exceeded the risk of cancer-attributed death in patients with an age-adjusted CCI > 3. In our experience, patients with a high risk of presenting non-tumoral death did not exceed the risk of cancer attributed death, with a CIR at 5 and 10 years of tumoral death of 22.7 and 30.5% in low risk patients, and 47.8 and 62.3% in high risk patients. These differences might be explained by the different characteristics of the patients included in the study of Mulcahy et al., that studied 230 patients with larynx cancer treated with total laryngectomy (42%), from which 191 patients (83%) were treated with surgery alone, and 318 patients (58%) treated with larynx preserving approaches [15].

In the treatment of LAHNC it is well accepted than IMRT decreases toxicity compared with conventional planning of the radiotherapy [11]. Moreover, some recent studies point to a potential increase in survival among patients treated with IMRT that might be explained by lower toxicity [12]. However, our study did not show any significant association between the treatment technique and toxicity or mortality. One potential explanation is that in our patients, the main objective was preservation of the parotid glands and not of the constrictors. Investigations to ascertain whether IMRT may decrease non-tumoral deaths in LAHNC are needed.

Finally, we attempted to ascertain a potential association between the intensification of RT and an increase in non-tumoral death. In some investigations, the application of CRT has been associated with an increase in the incidence of non-tumoral deaths, limiting the superior efficacy against induction therapy [7]. Our results have not shown any association between the incidence of non-tumoral death and the treatment regimen. It should be noted that many patients who were treated with induction CT were followed RTCTX, and recent publications have not shown an inferior toxicity with this regimen compared with CRT [33]. Moreover, mucosal toxicity showed in the univariate analysis a significant association with competitive death that was not confirmed in the multivariate analysis. Factors that influence mucositis, such xerostomía [34, 35] and oral microbiota are being investigated and may improve the prognostic of pacients in the next future [36].

This study has some limitations. One of the most important, derived from the retrospective nature of the study, is how to classify the causes of death. For instance, we considered death to be competitive mortality when patients were free of tumour. However, other authors might have not followed the same criteria. Moreover, it can be difficult to distinguish between death secondary to comorbidity and death related to chronic toxicity. Other limitations that should be considered are also derived from the retrospective nature of the study, especially regarding toxicity and comorbidity. Finally, HPV status was not considered because during most of the years included in the study, HPV status was not routinely studied in our centre.

## Conclusions

In summary, competitive causes of death may limit the effectiveness of RT intensification, showing a CIR of 17% at 5 years in high-risk patients (who were older and had moderate and high comorbidity). The results highlight the need of taking into account comorbidities and age in the clinical setting to consider intensification with RT and CT in patients with LAHNC, and to prompt investigation of how to incorporate this clinical factor into clinical trials to individualise which patients would most benefit from therapy intensification.

## Data Availability

The datasets used and/or analysed during the current study are available from the corresponding author on reasonable request.

## Abbreviations

CCI-HN: Cancer-specific comorbidity index for Head and Neck
CRT: Concomitant chemoradiotherapy
CT: chemotherapy
ICT: Induction chemotherapy
LAHNC: locally advanced head and neck cancer
RT: radiotherapy
RTCTX: Radiotherapy and cetuximab
